# Cases in Practice

**Published:** 1855-07

**Authors:** D. L. M’Gugin

**Affiliations:** Professor of Physiology and Pathology in the Iowa Medical Department


### IOWA MEDICAL JOURNAL.

VOL. II. KEOKUK, IOWA, JUNE AND JULY, 1855. NO. V.

### ORIGINAL COMMUNICATIONS.

### CASES IN PRACTICE.

BY D. L. M'GUGIN, M. D.

Professor of Physiology and Pathology in the Iowa Medical Department.

The attention of the profession was called some fifteen or twenty years since, to the propriety of the use of inhalations of iodine in phthisis, and there were many well-attested cases of its efficacy. This practice was doubtless suggested by the use, for a long time previous in pulmonary diseases, of the inhalation of the vapors of burning tar, which had become a domestic remedy; and reports of miraculous results were so frequent, and apparently so well sustained, that medical men were led to examine into its efficacy, and very many to prescribe it. The beneficial results following the the use of the warm water vapor in tonsillary inflammation, in the anginose variety of scarlatina and other inflammatory affections of the upper air passages, doubtless encouraged a trial in phthisis of the vapor of iodine, in the hope that from the well-known therapeutic powers of that remedy, in other modes of its exhibition, it would favor the absorption of tuberculous matter.

A plan for the administration of the iodine vapor, if I remember rightly, had just then been suggested in the Philadelphia "Journal of Medical Sciences," and an opportunity then offered to me for a trial of its virtues. The case was that of a clergyman, who had all the symptoms of phthisis. The physical evidences left not a doubt as to the existence of tubercles, and of course no hope was then entertained of his ultimate recovery. But his cough and other symptoms were so distressing that our thoughts were set actively in motion in the pursuit of some means of mitigation and relief. I seized upon the vapor of iodine, and proceeded at once to carry it out into practical fulfilment. For this purpose an apartment, the demensions of an ordinary bed frame, was temporarily partitioned off from that adjoining the one in which he lay, and a lounge placed into it, on which he reclined.

The fixture for the production of the vapor was made of a hoop of sheet iron ten inches in diameter, from the lower edge of which extended down strips, reveted on, to answer as legs and long enough so that when the hoop was raised from the stand, a spirit lamp could be introduced, while on the upper side a dish was to be placed half filled with warm water. Above, and over this, a vial was suspended, from the mouth of which a woolen thread protruded and hung below the base of the vial, constituting a kind of syphon, and from the lower end a drop was intended to fall every second into the water in the dish, in an active state of ebullition from the heat of the spirit lamp below and under it.

Very soon the small apartment was filled with vapor strongly impregnated with iodine, and when in this condition the patient was introduced into it and remained from twenty to thirty minutes. At first it was used but once a day, then twice, and after a time, because of the relief afforded, the patient asked that it be exhibited three times in that period.

In a few days after he began its use, his cough was less harrassing, the sputa diminished in amount, his breathing as yet not much slower was nevertheless easier, his voice improved to a tone of more compass and a better resonance, his appetite and digestion also amended.

A persistence in its use resulted in still further relief to the cough, the breathing now was slower, the muco-purulent matter diminished in amount, and the expectoration was now attended with but little effort, the soreness of the throat was relieved, the occasional rigors, fever, and colliquative sweats, had disappeared in a month, and his strength was so much improved as to enable him to leave the house and take exercise in the open air.

He yielded to the urgent solicitations of the friends of a deceased person to preach a funeral sermon, and a deep sense of religious obligation impelled him to undertake the task when the temperature was low and the atmosphere humid. The symptoms returned with increased violence; and although from that time he sank rapidly, yet he derived much relief from the use of the inhalation of the iodine vapor. When from prostration he could expectorate only with great difficulty, the vapor would stimulate the pulmonary power to greater activity, and expectoration would take place and temporary relief follow. The appliances for the production of the vapor were now brought within the curtains of the bed, and these closed.

Subsequent reflections upon this case have led me to hope that, had this exposure been avoided, the patient put upon the use of cod liver oil, and the vapor persisted in, that recovery was not impossible and a cure might have been affected. But we knew nothing of this valuable means of furnishing pabulum to the blood and nutrition to the tissues at that time. He was much emaciated, and such was the condition of the stomach that digestion was imperfect and therefore an insufficiency furnished for nutritive assimilation.

I have thus given the history of this case, which I have never since lost sight of, in the hope that others may be induced to make trial of the inhalation. It must be remembered, however, that I did not overlook the happy influences of water vapor at the time being conjoined with, and materially modifying, the iodine vapor. Where they can be used conjointly, it is better than when the iodine is used alone, as the water vapor dilutes the former, and there is usually less irritation produced upon the mucous lining of the bronchia. Recently, inhalations of vapor (and even in the form of finely levigated powders) has been introduced and is now practiced by many with manifestly good results.

I will now relate a case of more recent date, the history of which and the results, are not only interesting but very encouraging.

In December last, I visited a gentleman, about 40 miles distant in the country who had been confined to his house for some months previously. According to my notes, which I recorded at the time in my "visiting list," the following were his symptoms:

Marked dullness in the sub-clavicular and in the superior scapular regions. There is dullness indeed over the whole right breast, but particularly in the above localities. Normal resonance over left side except slight dullness in the clavicular portion. Auscultation shows loud mucous rale over the right breast, but under the clavicle there is gurgling and pectoriloquy. There are slight puerile manifestations on the left side. The muco-purulent expectoration was abundant and the cough troublesome. There had been rigors followed by fever, and occasionally these were succeeded by profuse sweats. There were some dyspeptic symptoms, but as yet no diarrhœa. The respiration was 30, and the pulse 100.

Dr. Baily, of Vernon, in this State, who felt a lively interest in this case, accompanied me to the residence of the patient, two miles distant from the village. He was fully satisfied of the correctness of the above record, and felt to regret that the disease had advanced so far as to be beyond probable recovery. Indeed few there are, who would have felt otherwise, for there was little prospect of relief from any course of medication, except the alleviation of the more painful symptoms. Such was the prevalent opinion, that upon my return to the village to partake of his hospitalities for the night, his intelligent lady remarked to me that "this time your journey through the cold will be of non-avail."

Hopeless as the case appeared, I nevertheless determined upon a thorough trial of the cod liver oil and the iodine inhalations. I ordered the oil in the usual doses, and directed that it should be each day increased in amount. A pure article was procured, and he commenced its use. I directed a sponge to be saturated with the tincture of iodine, and placed in a glass tumbler, and kept in the neighborhood of his face during the day and upon his pillow at night. In this way the air was qualified with its vapors all the while, and at each inspiration, he would inhale small portions. He was also directed to use a liniment prepared by triturating iodine and adding gradually cod liver oil until ultimately mixed. The formula was iodine ʒss to oil ℥i. This to be rubbed over the right breast once every day.

He used two bottles of the pure oil, and then procured two others which were found to be impure. The effect was manifestly very different from that produced by the first he had taken. The patient himself, under the use of the first, felt that he was gaining, but while taking the latter, he found himself retrograding. So soon, however, as he recurred to the use of a pure article, he immediately spoke of the change in his feelings. I mention this instance to show, as far as a single one will go, that the opinions entertained by some that there is as much remedial power in one oil as there is in another, are not well founded. The spurious oil above referred to, was doubtless lard oil freed from its stearine to which a little train oil was added to render it fishy in taste and smell.

He persevered faithfully in the plan of treatment, and slowly and steadily improved. The following extract of a letter dated April 3, 1855, from Dr. Baily, gives the following account of his case up to that time :

"I know you will be pleased to hear that your patient, Mr. Davis, is improving quite rapidly, and bids fair at present to enjoy pretty good health. He believes now that he will get well. He has used six large bottles of cod liver oil in addition to those he used before procuring the spurious article. His faith in it is so strong that he wishes me to procure him four bottles more. Every one acquainted with him is surprised at the improvement which has taken place in his case."

More recent intelligence is even more gratifying as he continues to improve, and is every day visiting his friends in the neighborhood and says he is nearly well, almost free from cough and all the other unfriendly symptoms.

Before concluding this article, which was chiefly designed to encourage the profession in the prosecution of the treatment of phthisis, even when from the aggravated character of the symptoms, any plan would promise but little. Bennett, in his excellent treatise upon tuberculosis, gives a number of cases in which there were recoveries under most unpromising symptoms. Dying subsequently from other diseases there were found in the lungs the traces of former tubercles by the cicatrices and the deposition of calcarious matter after absorption. These facts are sufficiently numerous to encourage to continued trial, however forbidding and unpromisnig the cases may present themselves.

A passing glance at the pathology of tuberculosis will show the propriety of the plan of treatment so strongly urged by Bennett, and others with the addition of inhalation of iodine vapor. It must be very brief, for our space or time will not permit a more lengthy discussion, although upon a subject of so much importance.

It is maintained that phthisis is hereditarily transmitted, and all experience is in proof of the position. It is said that the *predisposition* is transmissible, and here at the threshold of the investigation an enquiry will arise in what this predisposition consists, or in other words, what is it? This is called a hereditary quality, but what is it? Is it a simple proclivity or tendency to this disease? Is it an inherent quality, or is it that the whole organism is operated upon by exterior influences resulting in the production at the time of a certain class of phenomena? This may be said of many other ailments; but here is a disease to which all are not obnoxious, and yet transmissible in its quality. It is then *sui generis* and all are not alike predisposed to it. Predisposition to a disease lies in obeyance till an actor developes it. Small-pox is a class of diseases to which all are predisposed. We imbibe from our parents this predisposition. But Simon maintains that this hereditary quality in samll-pox is a *material*, which material lies latent until awakened into activity. This when once awakened, passes through such changes as to end in annihilation, and the individual no longer carries within him this predisposition. It has been expended, although inherited. This *affectio occulta* in ancient medical literature, is predisposition, and Henle maintains that "the pathological condition which is called predisposition, is *disease*," and here we would remark that a disease whose hereditary quality is so plainly marked as phthisis, and so often following in the line of descent, is predisposition of the strongest feature; and if "predisposition is disease," then the disease itself in an occult form is transmitted.

If, as Henle again says, "disease is a type according to which organic beings develope themselves," then we have here a predisposition to phthisis, which predisposition is itself disease. *Type* is form, *disease* is then form, and this brings us to the stand point which I take in the opinion formed of this disease, and that is, that we inherit the *predisposition*; this translated is *disease*; this again runs into *type*, and then into *form*. There is no *form* in proclivity, tendency or proneness; it applies *only* to material substances, *to matter*. It is my opinion, from a careful examination of the premises that we inherit a material in the blood when we inherit the predisposition to phthisis, just as much as we inherit a material to be operated upon by the small-pox contagion, the latter a common inheritance, the former a special one, but is almost as certain to be developed under certain existing causes.

Persons then are born with the *disease* and are *diseased* during life, and this may be extended through from the cradle to the grave, and yet not evince internal changes, but still will transmit the material to their offspring, whose different relations to the external world will exhibit changes such as their grand parents evinced in their lives.

This is a common sense view of the pathology of tuberculosis and is entitled to as much consideration as Simon's views in relation to the small-pox material inherited by the human family at birth. He maintains that small-pox, when once developed into activity, is the result of a change in that ever present morbid material in the blood, and that it does not result from some inherent tendency without *materiality*, *type* or *form*. Gravitation is an abstract consideration, and would not be known to exist without matter, nor would the attraction of cohesion be recognised as a law, unless particles existed to be united by it. The pustules of small-pox would not exist either, unless there was a material to be awakened into activity, resulting in consequences with as uniform changes, as the laws of gravitation and cohesion exert upon inorganized matter. This change extinguishes the predisposition by changing the character of the material, or by the actual loss of so much of the original organization as we lose the thymus gland because no longer necessary, but not in the same manner nor having the same results following.

The abstract fact of predisposition, as commonly understood namely that of tendency or proclivity, can be better applied as a term to an organ whose susceptibility to disease is influenced by the actual disease of another organ, as for instance the heart is prone to diseased action when the lungs are actually diseased. There is no hereditary quality in this instance over and above the common inheritance of mankind to all kinds of afflictions. But when we speak of the hemorrhagic diathesis, the rheumatic diathesis, we naturally couple with it the idea of some peculiar physical conformation. The term *diathesis*, as used and generally understood, is vague and ambiguous, and too much of the same quality with the term "sympathy," which answered well to retreat behind when hard pressed for a more satisfactory explanation.

But whether the tubercular material be a product of accidental formation, or pre-existed as an ever present material in the blood, it is nevertheless unlike the inflammatory product, and differs also from those which constitute the basis of other epigenic forms and growths. It is then *sui generis*, just as small-pox material is peculiar to itself and differing from all others.

Under what circumstances is it exuded? That state of the blood, which favors it, is when that fluid is deficient in its constituents, and when the proportions are abnormal. When the body is exhausted by disease or other causes, then it is that the blood is changed by a loss of its life-imparting constituents—then it is that exudation will take place more readily. The changes consequent upon maturity, and in the previous growth of the body, will also favor exudation.

Volumes have been written upon the prophylaxis of this disease in cases where there is a well-known *family predisposition*. Climate, diet, exercise, with medication have been each and all recommended and practiced to combat this *immaterial something*, this morbid predisposition, which terms, according to Henle's definition, are senseless and meaningless. The measures above indicated are *curative* and not prophylactic in the sense in which that term is used. They are measures active in the cure of the disease in a certain stage by holding the system above a condition in which exudation will take place. The next step in the malady is exudation and the deposition of tuberculous matter into tissues, whose organization is such that they more readily permit it to take place within them. But we avoid this still more unfriendly stage of this disease by preventing the exudation, and this brings us not only to consider the best means to prevent it, but which also offers us the best means of removal when it has taken place.

The best means of prevention is to preserve the normal constituents of the blood, and also to preserve if possible, the normal proportions. This done and there is less liability to exudation. The blood then must be supplied with its needed *pabulum*, and how is this to be accomplished? I answer by preserving, or re-

Establishing if lost, the nutritive and assimilative functions. This done and the staminal forces of the system will be established and preserved, and the blood prevented from falling into that unfortunate condition, in which advantage is given to the morbid material, always ready to take it in order to escape into the tissues whose condition permits its presence. What hygienic and dietetic course should be persued, would occur to any one at a glance.

But what course shall we pursue when exudation has already taken place? This question is more difficult to answer, and a problem much more difficult to solve. The indications in my opinion are two-fold. *First*, to prevent a further exudation: and *second*, to induce absorption of the material exuded.

The first then is to reform and repair the blood which has degenerated from its normal condition through the agency of malnutrition, and from an analysis of, and by experience with cod liver oil, it promises more than any other remedy. Its peculiar therapeutic action is not precisely known, but more than anything else yet known, it furnishes the requisite pabulum to the blood by which its richness is restored and its normal properties better established. It is maintained that it enters into the blood and plays an important part in its initiatory formation, and saves it from errors in its early developement, during which, in tuberculosis, its life is impaired by supplying nutrition and substance to the tubercular material which, in my opinion, is an inherent organization, and like any other organized tissue, looks for its supplies of nutrient material. The supply to this material will then be withdrawn, because the nutrition goes to the normal formation of the blood. The supply of tuberculous material for exudation is thus cut off, is not deposited, and the vital forces are restored. Now absorption will be quickened, and in the effort aid will be afforded by the iodine vapor which is inhaled from time to time. In this way the second indication will be met.

The diet should be nourishing, and a large share gleaned from the animal, in order to supply to the blood the requisites for its normal constituent. In cases of great laxity of fibre and tone, the preparations of cinchona may be used profitably, especially where there is loss of the tone of the stomach, attended with feeble digestive powers. It is all important to preserve the digestive powers in normal condition as far as possible, in order that we may derive all the required benefits of nutrition. This fact cannot, in my opinion, be too strongly pressed upon the attention of the practitioner. It is all important to keep up the dynamic forces and equally imperative to restore tone to the system where it has been lost or expended. Every effort should be exerted to this end, and this indication should not be forgotten for a single moment. If the stomach will not receive full doses of the cod liver oil, then it should be given in divided doses, in order that the amount be taken within the time. Minimum doses can be given with a stomachic, as the infus. cort. ort., aurant., or the weak solution of the ext. cinchona.

The vapor of iodine should be mingled with watery vapor if possible, in order to dilute it that it may not irritate the mucous lining of the bronchia too much at first. In this way, these tissues can be educated to bear it in greater intensity with impunity.

In conclusion, permit me to advise, that no case be abandoned to its own course however hopeless it may appear or however aggravated the symptoms.

